# An On-Orbit Dynamic Calibration Method for an MHD Micro-Angular Vibration Sensor Using a Laser Interferometer

**DOI:** 10.3390/s19194291

**Published:** 2019-10-03

**Authors:** Yingjie Wu, Xingfei Li, Fan Liu, Ganmin Xia

**Affiliations:** State Key Laboratory of Precision Measuring Technology and Instruments, Tianjin University, Tianjin 300072, China; wuyingjie666@tju.edu.cn (Y.W.); liufan@tju.edu.cn (F.L.); shenlongmingxia@126.com (G.X.)

**Keywords:** MHD, micro-angular vibration sensor, on-orbit dynamic calibration, sensitivity, laser interferometer, installation error

## Abstract

The magnetohydrodynamic (MHD) micro-angular vibration sensor is a significant component of the MHD Inertial Reference Unit (MIRU) and measures micro-amplitude and wide frequency angular vibration. The MHD micro-angular vibration sensor must be calibrated in orbit since the ground calibration parameters may change after lift-off. An on-orbit dynamic calibration method for the MHD micro-angular vibration sensor is proposed to calibrate the complex sensitivity of the sensor in high frequency. An absolute calibration method that combines a homodyne laser interferometer and an angular retroreflector was developed. The sinusoidal approximation method was applied, and the calibration system was established and tested using a manufactured MHD sensor. Furthermore, the measurement principle and installation errors were analyzed, including the eccentric installation error of the retroreflector, the tilt installation error of the retroreflector, and the optical path tilt error. This method can be realized within a rotation range of $\pm 3^{\circ }$ and effectively avoid the installation error caused by mechanical errors. The results indicate that the calibratable angular vibration frequency range is 25–800 Hz, and the angular velocity range is 0.076–7590 mrad/s. The expanded uncertainties of the sensitivity amplitude and phase shift of the calibration system for the MHD micro-angular sensor are 0.04% and $1.2^{\circ }$ (k=2).

## 1. Introduction

### 1.1. Background

High-resolution Earth observation remote sensing satellites, deep space exploration and remote sensing spacecraft, and deep space laser communication satellites have extremely high requirements for the attitude accuracy and stability of both the spacecraft and its payload. When a spacecraft stably operates in orbit, micro-angular vibration will inevitably be induced by external factors, such as the opening drive of the stretching mechanism, the docking and separation of the cabin, and solar radiation pressure. A pointing accuracy of less than 1 μrad is usually required for the attitudes of high-resolution remote sensing satellites, which are susceptible to micro-angular vibrations that can cause serious interference and reduce image quality. The Hubble Telescope, launched in 1990, failed to work after orbit injection as a result of the tremor response, with a maximum frequency of 500 Hz and a minimum amplitude of 1.5″ [1]. Therefore, it is crucial to effectively measure the micro-angular vibration motion, which can be further eliminated by utilizing a vibration isolator or an active vibration suppression apparatus.

Micro-angular vibration is typically characterized by a small amplitude (<1″) and a wide frequency band (0–1000 Hz). The vibration energy is large, with a frequency that ranges from several to several hundred hertz, and difficult to attenuate [2]. The magnetohydrodynamic (MHD) micro-angular vibration sensor has the advantages of high bandwidth, low noise, and small volume, and it is a relatively mature micro-angular vibration sensor for satellite platforms [3]. For example, the NASA Lunar Laser Communication Demonstration (LLCD) system, developed in 2013, uses the MHD micro-angular vibration sensor as an important inertial component to maintain a stable positioning platform, which is capable of achieving a pointing accuracy of less than half an inch between the ground and the Moon [4,5].

Nevertheless, the MHD micro-angular vibration sensor is likely to encounter parameter variation when on-orbit micro-angular vibration is measured. The Orbital Acceleration Research Experiment (OARE) has shown that the ground calibration parameters of inertial devices, such as ultra-sensitive accelerometers, can vary significantly after the satellite is launched, and periodic calibration is necessary during its flight [6]. When the MHD micro-angular vibration sensor enters the space environment and is subjected to conditions such as a vacuum and ultra-low temperature, its operating state changes as the magnetic fluid properties change. Thus, the sensor should be calibrated before normal operation. It is difficult for ground-based simulation equipment to fully reproduce the temperature, humidity, and weight in space. A slight difference can have a great impact on the performance of the micro-angular vibration sensor. Therefore, it has become essential to dynamically calibrate the MHD micro-angular vibration sensor in orbit.

An on-orbit calibration method can also be applied to the initial alignment of the inertial system. The ATA Company built a new-generation MIRU by combining the MHD micro-angular vibration sensor, microelectromechanical system (MEMS) gyroscope, and quartz accelerometer. The measurement error of the inertial measurement unit (IMU) affects the resolution of the attitude, velocity, and position of the inertial navigation system through integration or differentiation. Consequently, the initial alignment of the MHD micro-angular vibration sensor is a necessary initial condition for the inertial navigation system before it enters the navigating operation state. The calibration of the sensor’s complex sensitivity is a key component of the initial alignment [7]. In particular, the accuracy of the calibration can directly affect the initial alignment error and attitude of the payload.

### 1.2. Methodology

There are diverse and mature methods for calibrating angular vibration sensors in the laboratory. According to the principle on which they operate, these methods can be divided into absolute calibration methods and relative calibration methods. An absolute calibration method calibrates sensors using a basic quantity in nature, while a relative calibration method calibrates the parameters of the target sensor by using the output signal of a standard sensor under the same vibration conditions. In this section, we explore and evaluate existing calibration methods of on-orbit dynamic calibration to establish the most appropriate approach.

First, absolute calibration methods based on a laser interferometer are introduced. In 1998, Physikalisch–Technische Bundesanstalt (PTB) combined homodyne and heterodyne technology with a diffraction grating target for the first time to establish an angular vibration calibration system based on the sinusoidal phase diffraction grating of holographic manufacturing [8]. In 2000, PTB concluded that the sinusoidal approximation method was superior to other interference signal processing methods, such as the minimum point method and the fringe counting method [9]. In the same year, the International Organization for Standardization (ISO) established a laser interferometer calibration system with sinusoidal excitation, for which a series of indicators were stipulated [10]. In 2006, the China Changcheng Institute of Metrology and Measurement (CIMM) developed a standard device to calibrate angular acceleration. The device uses a diffraction grating and two heterodyne laser interferometers to measure the angular vibration of an angular vibration table [11,12]. In 2006, the Korea Research Institute of Standards and Research (KRISS) proposed a laser interferometer based on an “angle prism”, which combines the prisms and retroreflector as targets for the beam [13]. In 2014, a new type of heterodyne laser interferometer based on a diffraction grating was developed [14]. In 2016, a dual differential laser Doppler angular vibration method based on a ring laser was proposed [15]. A detailed comparison of these methods and their key parameters are given in Table 1.

Next, relative calibration methods are summarized. A relative calibration method generally requires that the accuracy or dynamic performance of a standard sensor is the same as or better than that of the sensor being calibrated. As an angular velocity sensor, the output signal from a gyroscope is suitable for calibrating the relevant parameters of MHD micro-angular vibration sensors. New gyroscopes with high precision and reliability include laser gyroscopes, fiber optic gyroscopes, and MEMS gyroscopes. A relative calibration method was proposed for measuring angular displacement motion by utilizing a novel time grating sensor [16]. The time grating sensor can perform indirect angle measurement by converting a difference in spatial displacement into a time difference in a locomotor reference system. This process is implemented by establishing a reference system of “constant velocity” motion inside the sensor. However, the literature has indicated that the accuracy of the time grating sensor varies substantially at high frequencies [17]. The characteristics, principles, advantages, and disadvantages of these relative calibration methods are summarized in Table 2.

In addition to the above-described absolute calibration and relative calibration methods, a few other methods based on other principles have been developed. For example, linear acceleration can be converted to angular acceleration for measurement purposes; such a method was developed by ENDEVCO Company in the United States. In the literature, a free-oscillation method for calibrating the parameters of an angular accelerometer based on the principle of the torsion pendulum was established [18]. In 2016, the National Institute of Metrology of Japan (NIMJ) proposed an angular velocity calibration system with a self-calibratable rotary encoder [19]. The characteristics, principles, advantages, and disadvantages of other calibration methods are summarized in Table 3.

An on-orbit calibration system generally requires a high calibration efficiency, convenient operation, and small size. The MHD micro-angular vibration sensor requires high calibration accuracy and a wide passband: the order of magnitude of the angular resolution can be as high as μrad, and the measurable frequency can reach or exceed 800 Hz [20]. Table 1, Table 2 and Table 3 report each method’s “priority”, which combines the method’s convenience, size, accuracy, coverage frequency, and additional advantages and disadvantages. The greater the number of stars in the last column, the higher the “priority” of the method. Notably, the methods in the three tables that have two or three stars are all absolute calibration methods, which are more suitable for calibrating MHD micro-angular vibration sensors. In particular, the target of the retroreflector can avoid installation error and is more suitable for measuring micro-angular vibration compared with a diffraction grating. The drawback of this method is that the retroreflector takes up more space than is preferable. The sinusoidal approximation method can then be used for processing the interference signal to simultaneously calibrate the amplitude and phase, with the advantage of suppressing the extent of the disturbance.

In summary, dynamic calibration with a combination of a homodyne interferometer and a retroreflector is suitable for calibrating the MHD micro-angular vibration sensor. More specifically, the calibration is performed by the sinusoidal excitation method and sinusoidal approximation method. In this study, the system was constructed, and experiments were conducted on the calibration system and MHD micro-angular vibration sensor to evaluate the system performance.

## 2. Methods and Principle

### 2.1. System Configuration

**Figure 1:**
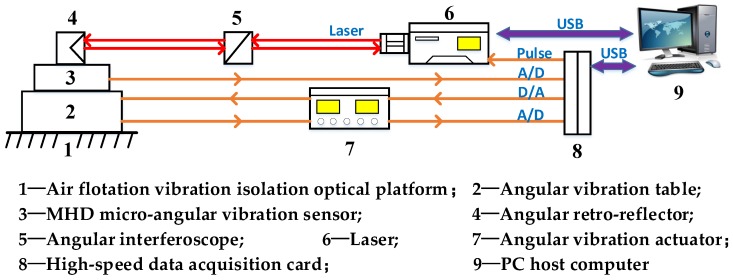
Structure of the calibration system.

**Figure 2:**
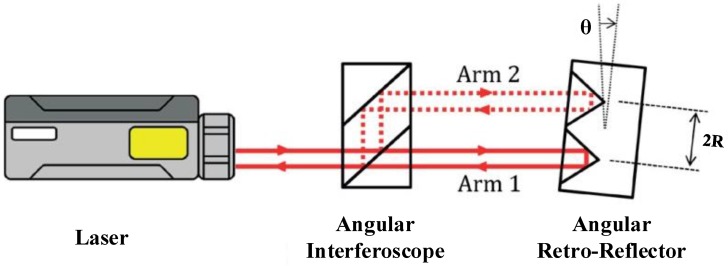
The measuring structure of double-beam angular vibration.

The structure of the calibration system is shown in Figure 1. The main components of the calibration system are the angular vibration excitation system and the homodyne laser interference calibration system. The angular vibration excitation system consists of an angular vibration table and an angular vibration actuator, and the homodyne laser interference calibration system consists of a laser, an angle interferoscope, and an angular retroreflector. As shown in Figure 2, the two-beam differential structure can increase the quantization interval of the interference by two-fold compared with the single-beam measurement structure. As a result, the resolution ability is improved. The angular retroreflector and the MHD micro-angular vibration sensor are fixed to the angular vibration table by a clamp, which enables the same angular vibration state to be achieved. During calibration, the host computer controls the angular vibration table’s generation of sinusoidal angular motion through a data acquisition card and an angular vibration actuator. The output voltage signal of the MHD micro-angular vibration sensor and the output angle signal of the laser interferometer are acquired synchronously. Then, data processing is performed in LabVIEW, and information such as complex sensitivity can be obtained.

Since the angular velocity is the physical quantity directly measured by the sensor, the angle signal obtained by laser interference needs to be differentiated and then converted to an angular velocity signal for further processing. Information such as amplitude and phase shift can be obtained by applying the Fourier transform to the two signals separately. Changing the frequency in repeated experiments generates the frequency response curve of the sensitivity.

The sensitivity amplitude S and phase shift ΔΦ of the MHD micro-angular vibration sensor are calculated as shown in Equations (1) and (2). In these equations, $V_{MHD}$ and $\varphi_{MHD}$ are the amplitude and phase of the output voltage of the sensor, and Ω and $\varphi_{\Omega }$ are the amplitude and phase of the angular velocity measured by the laser interferometer under the same vibration.

(1) $$S=\frac{V_{MHD}}{\Omega }$$

(2) $$\Delta \Phi =\varphi_{MHD}-\varphi_{\Omega }$$

### 2.2. Theoretical Background

In this section, the principle of angular vibration measurement based on the Michelson interferometer is introduced, and the effect of standard angular vibration excitation on calibration performance is analyzed.

#### 2.2.1. Michelson Laser Interference Principle and the Sinusoidal Approximation Method

**Figure 3:**
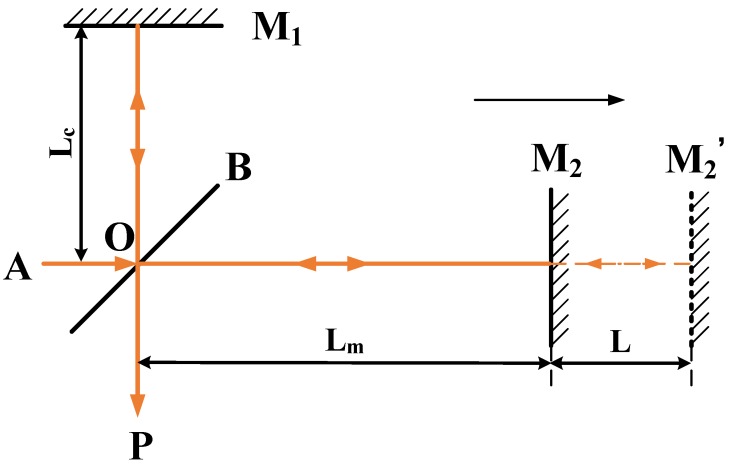
Michelson interferometer.

The interference method with a retroreflector as a target is based on the Michelson interferometer. As shown in Figure 3, the laser beam incident from point A is split into two laser beams when it passes through the beam splitter B. Subsequently, the two beams return to point O along the original path after they pass through the reference retroreflector $M_{1}$ and the target retroreflector $M_{2}$ in sequence. At point O, interference occurs, and the beams reach the photoelectric detector P. The photodetector can detect a change in light intensity and generate a photocurrent. In particular, a change in the magnitude of the current contains the relevant movement information, from which the amplitude and phase can be acquired by the sinusoid approximation method.

When the target retroreflector moves from $M_{2}$ to ${M_{2}}^{\prime }$, the change in the optical path difference Δ of the two beams can be calculated using Equation (3).

(3) $$\Delta =\Delta_{2}-\Delta_{1}=2n\left(L_{m}+L-L_{c}\right)-2n\left(L_{m}-L_{c}\right)=2nL$$

where *n* is the refractive ratio of air, $L_{m}$ denotes the initial distance from the target retroreflector $M_{2}$ to the splitting point O, $L_{c}$ denotes the distance from the reference retroreflector $M_{1}$ to the splitting point O, and L is the moving distance of the moving retroreflector $M_{2}$ along the direction of the optical path.

If it is assumed that ${M_{2}}^{\prime }$ is conducting a stable sinusoidal oscillation at the equilibrium position, where the optical path difference Δ varies according to Equation (4), then

(4) $$\Delta =2nl=2n\left[l_{0}+a\cos\left(\omega^{\prime }t+\varphi^{\prime }\right)\right]$$

where $l_{0}$ is the distance between the equilibrium position and the initial position. Moreover, *a*, $\omega^{\prime }$, and $\varphi^{\prime }$ are the amplitude, angular frequency, and phase of the rectilinear vibration, respectively.

The photodetector is used to detect the intensity of the interfering light, but it cannot detect the ultra-high frequency component, and the DC signal component could be ignored. The alternating value of the photovoltage U can be expressed by Equation (5).

(5) $$U=kE_{a}E_{b}\cos\Delta \varphi =kE_{a}E_{b}\cos\left(\frac{2\pi }{\lambda }\cdot \Delta \right)=kE_{a}E_{b}\cos\left[\frac{4an\pi }{\lambda }\cos\left(\omega^{\prime }t+\Phi^{\prime }\right)+\frac{4n\pi }{\lambda }l_{0}\right]$$

where *k* denotes the gain coefficient, $E_{a}$ and $E_{b}$ denote the electric field amplitudes of the two beams, Δφ denotes the phase difference, and λ denotes the wavelength of the light in the air.

If the beam passes through the phase-shifting optics before it reaches the photodetector, two orthogonal photoelectric signals can then be obtained and solved by the sinusoidal approximation method. By dividing two output voltage signals and determining the arctangent, we obtain Equations (6) and (7).

(6) $$\varphi^{\prime \prime }=\arctan\frac{U_{2}\left(t\right)}{U_{1}\left(t\right)}+n\pi$$

(7) $$\varphi^{\prime \prime }=\frac{4an\pi }{\lambda }\cos\left(\omega^{\prime }t+\varphi^{\prime }\right)+\frac{4n\pi }{\lambda }l_{0}$$

The curve described by Equation (6) is fitted by using the least squares method, and the values of *a*, $\omega^{\prime }$, and $\varphi^{\prime }$ can be obtained by combining Equation (7) with Equation (6). As a result, the main vibration information is retrieved.

#### 2.2.2. Principle of the Angular Vibration Excitation System

In the ISO standard, the recommended excitation methods are primarily an electric vibration exciter and angular vibration exciter based on a brushless motor. The most commonly used small-volume angular vibration exciter is the brushless motor. A brief description of the operating principle of the motor and the analysis of factors that potentially affect the calibration accuracy are presented below.

**Figure 4:**
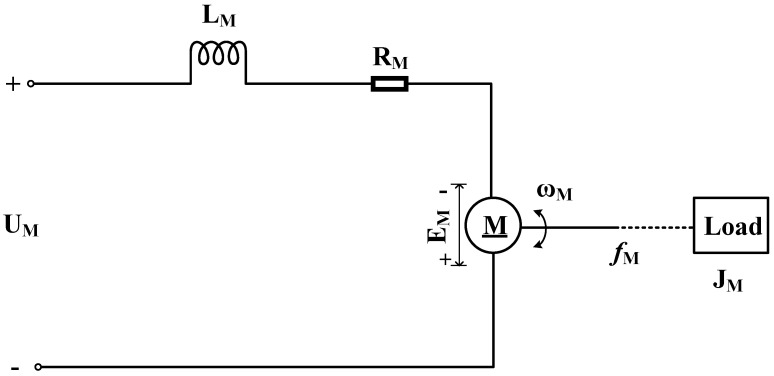
A simplified model of the motor carrying a load.

The motor works according to the law of electromagnetic induction and can convert the input electrical energy into mechanical energy. As shown in Figure 4, the input voltage $U_{M}\left(t\right)$ can generate electromagnetic torque through the motor M to drive the rotation of the load. When the load torque is not considered, the relationship between the motor speed $\omega_{M}\left(t\right)$ and the input voltage $U_{M}\left(t\right)$ can be described by Equation (8).

(8) $$L_{M}J_{M}\frac{d^{2}\omega_{M}\left(t\right)}{dt^{2}}+\left(L_{M}f_{M}+R_{M}J_{M}\right)\frac{d\omega_{M}\left(t\right)}{dt}+\left(R_{M}f_{M}+C_{M}C_{e})\cdot \omega_{M}\left(t\right)\right.=C_{M}U_{M}\left(t\right)$$

In this equation, $R_{M}$ is the resistance of the armature circuit, $L_{M}$ is the inductance of the armature circuit, $J_{M}$ is the equivalent moment of inertia of the motor and load folded onto the motor shaft, $f_{M}$ is the equivalent viscous friction coefficient on the motor shaft, $C_{M}$ is the motor electromagnetic torque coefficient, and $C_{e}$ is the counter-electromotive force coefficient.

Equation (8) specifies that the ideal motor is a linear time-invariant system. However, in practice, the nonlinearity of circuit components and mechanical systems can result in waveform distortion of the output angular vibration. Therefore, with high-frequency angular vibration, the influence of load inertia on the magnitude of the system output angular velocity and the total harmonic distortion (THD) must be adequately considered if the load-carrying capacity of the angular vibration excitation system is limited.

During the calibration process, the amplitude and phase obtained by fitting the sinusoidal parameters will be affected by the THD generated by the exciter. Studies have shown that the fitting error of the relevant parameters will increase as the THD increases [21]. Therefore, in practical experiments, closely monitoring the THD of the excitation system is indispensable. The best calibration results can be achieved when the distortion rate is much less than 2%.

### 2.3. System Error

In this section, the principle error is provided, and measurement errors are analyzed to evaluate the installation error due to the misalignment of the on-orbit calibrated optical path. The measurement errors discussed are those introduced by the eccentric installation of the retroreflector, the tilting installation of the retroreflector, and the optical path tilt.

#### 2.3.1. Principle Error

From the Michelson interferometer principle, it is known that when the retroreflector is used as a target, the measured optical path difference is the distance that it moves in the direction of the optical path. Thus, the distance that the mirror moves in a short time can be used to represent the corresponding arc length of the corner when the angular vibration is measured by the retroreflector method. In the following, the principle-induced errors within the maximum angular measurement range are evaluated.

**Figure 5:**
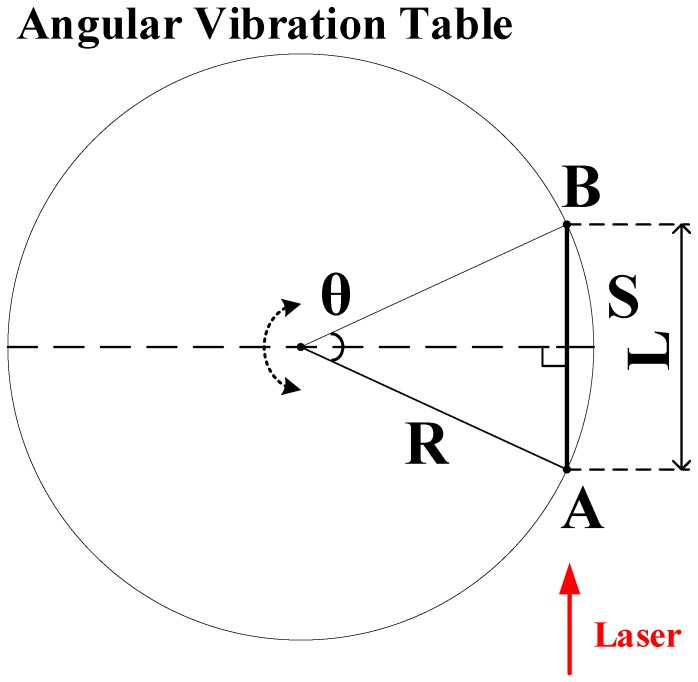
Angle measuring principle.

As shown in Figure 5, the mirror can be considered a mass point that is fixed at point A. Within time Δt, the angular vibration table rotates counterclockwise by angle Θ when the mirror moves from point A to point B. R is assumed to be the distance from point A to the angular vibration center, *L* is the linear distance from point A to point B along the optical path, and *S* is the arc length from point A to point B. The measured length principle error is described by Equation (9).

(9) $$\Delta s=s-l=R\Theta -2R\sin\frac{\Theta }{2}$$

Hence, if $\Theta =\pm 3^{\circ }$ and R=15 mm, then $\Delta s=\pm 8.971\times 10^{-5}$ mm, and the angle relative error $\frac{\Delta s}{s}=\pm 0.011\%$. On account of the small size of this error, its influence can be ignored within a rotating angle range of $\pm 3^{\circ }$.

#### 2.3.2. The Eccentric Installation Error of the Angular Retroreflector

**Figure 6:**
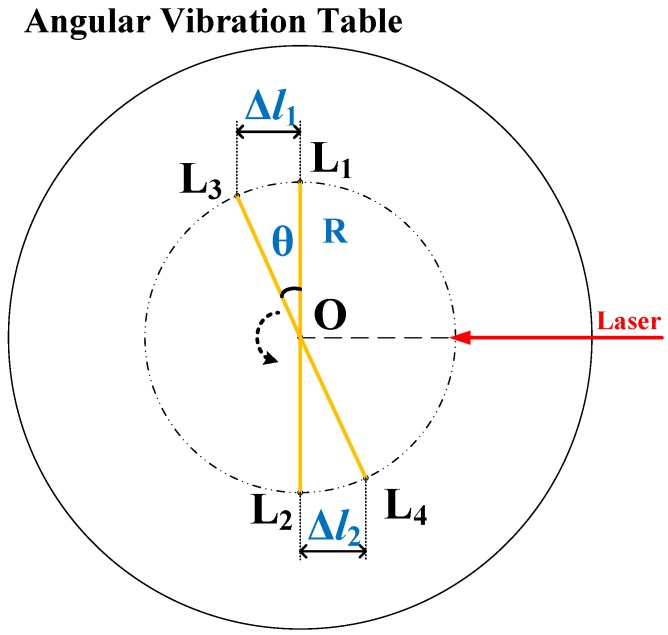
The standard installation of the angular retroreflector.

As shown in Figure 6, the standard installation of the angular retroreflector requires that the center of the retroreflector coincides with the center of the angular vibration table. The two rectilinear retroreflectors are simplified as point $L_{1}$ and point $L_{2}$. The laser beam is perpendicular to $L_{1}L_{2}$, and the distance between $L_{1}$ and $L_{2}$ is 2R. When the angular vibration table rotates counterclockwise by the angle θ within a period of time Δt, the retroreflectors move from points $L_{1}$ and $L_{2}$ to points $L_{3}$ and $L_{4}$, respectively. In this case, the sum of the distances Δl of the two mirrors moving in the direction of the optical path is defined as the optical path difference of the laser beam change, as shown in Equation (10).

(10) $$\Delta l=\Delta l_{1}+\Delta l_{2}=2R\sin\theta$$

When the angular retroreflector is eccentrically installed on the table or if the center of the installation position and the real center of vibration do not coincide, the measuring angle calculated by Equation (10) will deviate from its true value. Therefore, the effect of the error on the calibration result needs to be evaluated.

**Figure 7:**
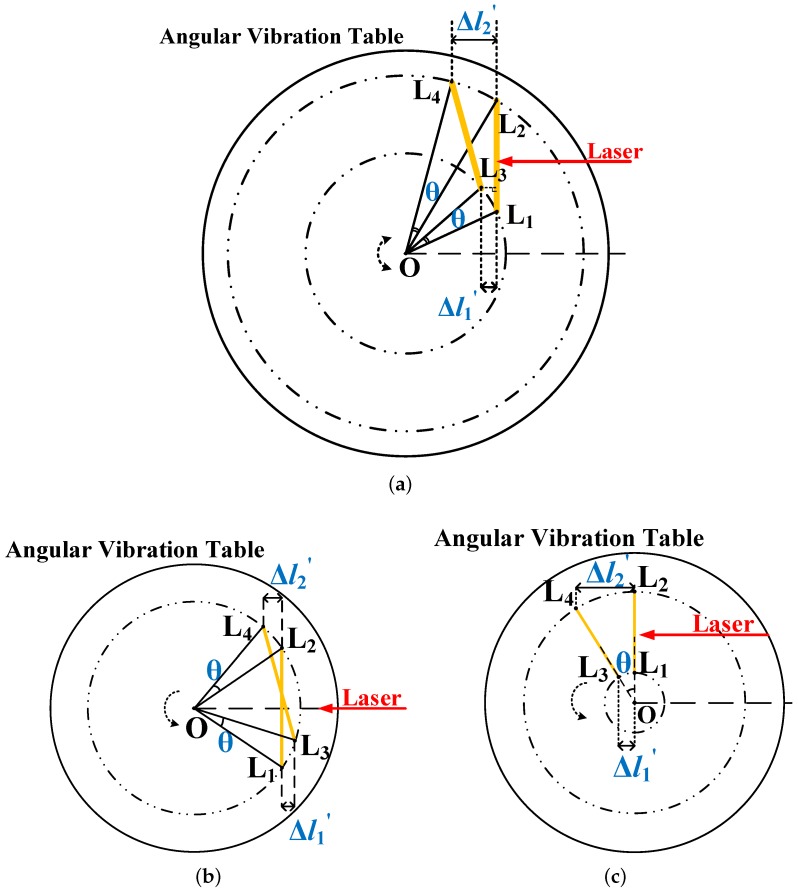
Eccentric installation of the angular retroreflector (**a**) when the angular retroreflector is completely located in the first quadrant, (**b**) when the angular retroreflector is translated a distance from the center position in the opposite direction of the optical path incidence, and (**c**) when the angular retroreflector is translated from the center position by a distance along the vertical direction of the optical path and $L_{1}$ is above point O.

For eccentric installation, as shown in Figure 7, the Cartesian coordinate system is established (with O as the center) and takes the negative incidence direction of the laser as the x-axis. The coordinates of each point are assumed to be $L_{1}\left(x,y\right)$, $L_{2}\left(x,y+2R\right)$, $L_{3}\left(x_{3},y_{3}\right)$, and $L_{4}\left(x_{4},y_{4}\right)$. $L_{3}$ and $L_{4}$ can be obtained from $L_{1}$ and $L_{2}$ by the rotating angle of θ, where $L_{3}$ and $L_{4}$ are described by Equations (11) and (12), respectively.

(11) $$\left(\begin{array}{c} x_{3}\\ y_{3} \end{array}\right)=\left(\begin{array}{cc} \cos\theta & -\sin\theta \\ \sin\theta & \cos\theta \end{array}\right)\left(\begin{array}{c} x\\ y \end{array}\right)$$

(12) $$\left(\begin{array}{c} x_{4}\\ y_{4} \end{array}\right)=\left(\begin{array}{cc} \cos\theta & -\sin\theta \\ \sin\theta & \cos\theta \end{array}\right)\left(\begin{array}{c} x\\ y+2R \end{array}\right)$$

With the solution obtained for the coordinates of these two points, the coordinates of $\vec{L_{3}L_{1}}$ and $\vec{L_{4}L_{2}}$ can be obtained. With eccentric mounting, the measured optical path difference $\Delta l^{\prime }$ can be calculated, as shown in Equation (13).

(13) $$\Delta l^{\prime }=\Delta l_{1}^{\prime }+\Delta l_{2}^{\prime }=\left|x-x\cos\theta +y\sin\theta |+|x-x\cos\theta +\left(y+2R\right)\sin\theta \right|$$

To illustrate the weighing of the error, three situations are considered, as shown in Figure 7.

1. When the angular retroreflector is completely located in the first quadrant, the measuring retardation can be represented as $\Delta l^{\prime }=2x\left(1-\cos\theta \right)+2y\sin\theta +2R\sin\theta$.
  If $\theta =3^{\circ }$, R=15 mm, and $L_{1}\left(1,1\right)$ are assumed, then $\Delta l^{\prime }=1.677$ mm.
  Then, the relative angular error induced by eccentricity is $\Delta \theta =\pm \frac{\theta -\arcsin\left(\frac{\Delta l^{\prime }}{2R}\right)}{\theta }=\pm 46.6\%$.
2. When the angular retroreflector is translated a distance from the center position in the opposite direction of the optical path incidence, the measured optical path difference can be expressed as $\Delta l^{\prime }=2R\sin\theta$.
3. When the angular retroreflector is translated from the center position by a distance along the vertical direction of the optical path and $L_{1}$ is above point O, the measured optical path difference can be expressed as $\Delta l^{\prime }=2y\sin\theta +2R\sin\theta$.

It is assumed that the angular retroreflector is designed to be installed at the center position, but the mirror is shifted by 0.1 mm in the positive direction of both the x-axis and y-axis as a result of mechanical positioning errors, among other factors. In other words, the condition is $\theta =3^{\circ }$, R=15 mm, and $L_{1}\left(0.1,-14.9\right)$. Then, $\Delta l^{\prime }=2R\sin\theta =\Delta l$ can be obtained.

In sum, the following two conclusions can be drawn. When the angular retroreflector is eccentrically mounted at any position on the angular vibration table, a large relative measurement error will result, except for the special case b. When the angular retroreflector is designed and installed at the center of the angular vibration table, the measurement results are accurate, even if something causes the retroreflector to be off-centered by a small distance.

#### 2.3.3. The Tilt Installation Error of the Angular Retroreflector

**Figure 8:**
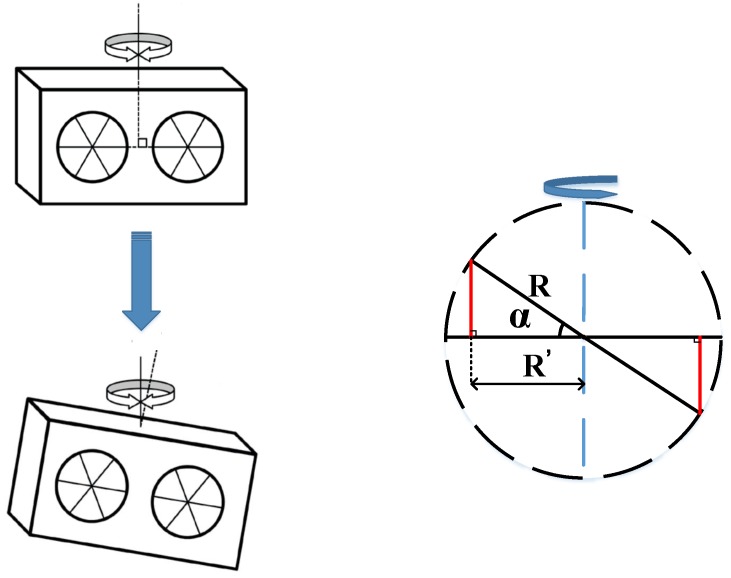
Tilt installation of the angular retroreflector.

When the angular retroreflector is aslant mounted on a plane, as shown in Figure 8, the angle between the angular retroreflector and the horizontal is α. This will cause the horizontal distance between the position in which the actual interference occurs and the vibration axis to change from *R* to $R^{\prime }$. The measurement optical path difference $\Delta l^{\prime }$ is expressed by Equation (14).

(14) $$\Delta l^{\prime }=2R^{\prime }\sin\theta =2R\cos\alpha \sin\theta$$

If $\alpha =1^{\circ }$, $\theta =3^{\circ }$, R=15 mm are assumed, then the relative angular error will be Δθ=±0.015%.

#### 2.3.4. The Tilt Installation Error of the Optical path

**Figure 9:**
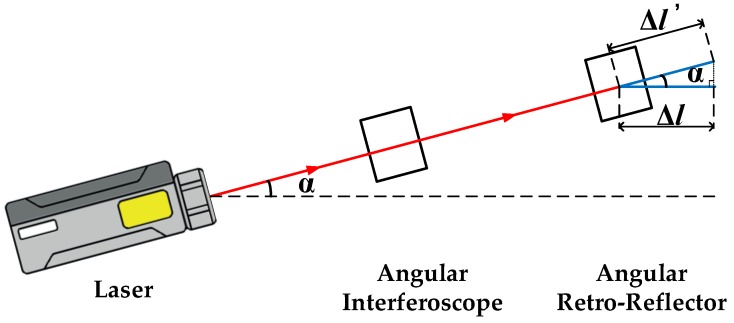
Tilt installation of the light path.

If the optical table and the optical components are not leveled strictly by the spirit level, then it is possible that the entire optical path will be tilted. As shown in Figure 9, the angle between the inclined light path and the horizontal is α, and the actual measured optical path difference is changed from Δl to $\Delta l^{\prime }$. The optical path difference $\Delta l^{\prime }$ resulting from the optical path tilt is expressed in Equation (15).

(15) $$\Delta l^{\prime }=\frac{\Delta l}{\cos\alpha }=\frac{2R\sin\theta }{\cos\alpha }$$

If $\alpha =1^{\circ }$, $\theta =3^{\circ }$, R=15 mm are assumed, then the relative angular error will be Δθ=±0.015%.

## 3. Results

### 3.1. Experimental Setup

**Figure 10:**
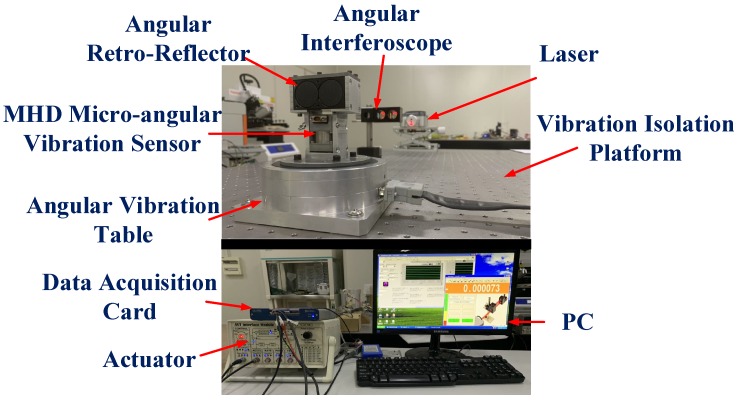
Calibration experiment configuration.

As shown in Figure 10, the main experimental instruments are the 105-AVT angular vibration table (Acutronic Company, Bubikon, Switzerland), the XL-80 laser interferometer (Renishaw Company, Gloucestershire, UK), the NI-6361 data acquisition card, the ZDT-P-MOT-F air flotation vibration isolation optical platform (Liansheng Company, Nanchang, China), and the PC host computer.

The signal from the data acquisition card and optical laser are collected simultaneously by applying the trigger-acquisition method to the system. When feeding a counter pulse into the fast pulse port of a laser with a data acquisition card, the card and the laser start to collect the signal from the falling edge of the pulse at the same time; this ensures that the amplitude and phase of the same signal are obtained. The vibration isolation platform in the system can provide a stable horizontal datum and isolate the vibration from the ground. Thus, the extra vibration will not influence the measuring result.

The experimental environment should be checked before the experiment. Because of the strict environmental requirements of a homodyne laser interferometer, the temperature and humidity must be stable. Moreover, the environment should be free of air turbulence. A stable environment can improve the relative homogeneity of laser wavelengths in the two measuring arms. When adjusting the optical path, the precision lifting platform and the spirit level can ensure the level of all optical instruments.

The frequency passband of the 105-AVT angular vibration table can be as high as 2500 Hz. The system uses an AC brushless motor, in which there is only the unit negative feedback with a compensation link added to the feedback circuit. Nevertheless, the parameters of calibration system need to be exceeded under non-reference conditions. It is not easy to directly set the amplitude of angular vibration by varying the input voltage. The 105-AVT angular vibration table is small in size and has a THD of less than 2%, which is suitable for installation on a spacecraft. However, its load-carrying capacity is poor. For high-frequency vibration, experiments must be designed to investigate the influence of the additional load installed on the table surface on the output angular acceleration of the angular vibration table. Whether the increase in the load inertia will amplify the nonlinear characteristics of the mechanical system and cause an increase in the THD of the angular vibration should also be studied.

**Figure 11:**
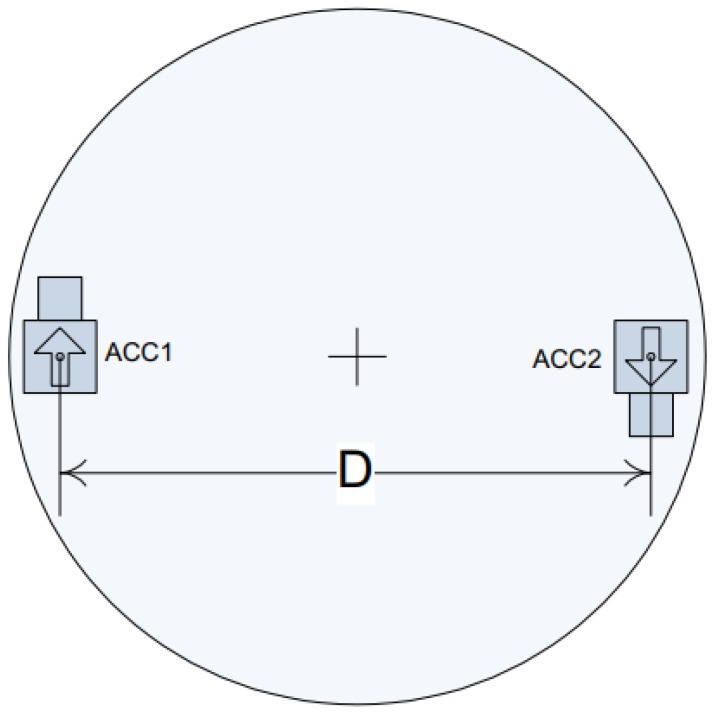
Accelerometer mounting diagram of the angular vibration table.

As shown in Figure 11, two linear accelerometers are configured under the table-board of the angular vibration table. When the average linear acceleration of the two accelerometers is determined, the converted angular acceleration can be used as a reference to compare with the measurement result of the laser interferometry calibration system. Since the accelerometers are not calibrated by the absolute calibration method and installation errors may exist, the output angular acceleration obtained directly from the angular vibration table can only be used as a reference signal rather than a standard signal.

**Figure 12:**
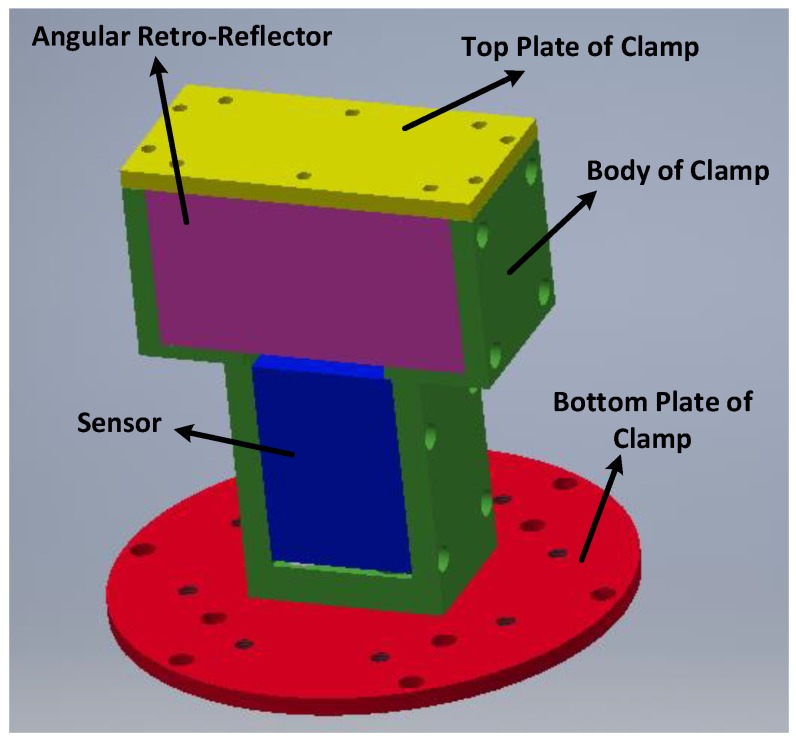
Schematic diagram of the clamp design.

When establishing the system, one of the main issues to resolve is whether the MHD micro-angular vibration sensor and the angular retroreflector can experience the same angular vibrations. Therefore, these instruments must be fixed securely to ensure measurement accuracy. In Figure 12, the pink cuboid represents the angular retroreflector, and the blue cuboid represents the MHD micro-angular vibration sensor. The middle part of the clamp is designed as one piece, and the MHD micro-angular vibration sensor and the angular retroreflector are fixed to the side of the clamp. Thus, inconsistent vibration caused by complicated mechanical transmission characteristics can be avoided when the screws are connected with different parts.

### 3.2. Experimental Results

As described in this section, a consecutive three-part experiment was designed to test the performance of the system by verifying the carrying capacity of the excitation system, acquiring the effective range of the calibration system, and calibrating the relevant parameters of the MHD micro-angular vibration sensor. The repeatability and nonlinearity of the key parts were tested. The micro-angular vibration calibration capability of the system was determined, and the uncertainty was assessed. The results are detailed below.

#### 3.2.1. Frequency Response and Distortion of the Excitation System Carrying Different Loads

For the purpose of qualitatively analyzing the influence of load inertia variation on the output angular acceleration of the excitation, two uniform disks were fixed to the table, and then the motor current and output angular acceleration were measured. The carrying capacity of the angular vibration table is about 1 kg. The two disks have the same mass and inertia of 554.88 g and $6.93\times 10^{-4}$ kg·m${}^{2}$, respectively. The output angular acceleration of the accelerometer inside the table was used as a reference to analyze the results.

**Figure 13:**
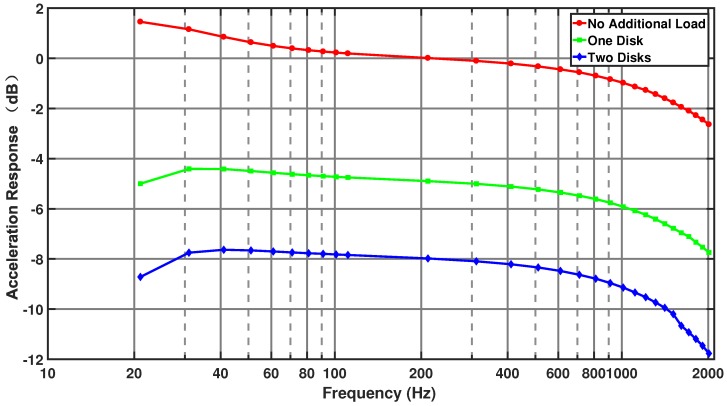
Angular acceleration response of the angular vibration table carrying different loads.

Figure 13 shows the output angular acceleration response curve for a frequency range of 20–2000 Hz when the angular table carries different loads. The response is the ratio of the output voltage signal of the accelerometer to the excitation voltage signal, with the unit converted to decibels. In Figure 13, the angular acceleration response of the excitation system at 2000 Hz is −2.6 dB without additional load. As the installed load inertia increases, the acceleration response of the excitation system decreases significantly. When the load inertia increases by $6.93\times 10^{-4}$ kg·m${}^{2}$, the ratio decreases by around 5 dB; when the load inertia increases by $13.86\times 10^{-4}$ kg·m${}^{2}$, the ratio decreases by about 8 dB. The decreasing trend of the response curve is greater at high frequencies.

Table 4 shows the THD of the motor current and the angular acceleration output signal in a frequency range of 20–2000 Hz when the angular table carries different loads. As shown in Table 4, the maximum THD of the motor current and angular acceleration are 0.049% and 0.340%, respectively. The THD increases as the frequency increases. The THD introduced by the nonlinearity of motor mechanic structure is much larger than the THD introduced by the nonlinearity of the motor equivalent circuit. The maximum THD of the motor current and angular acceleration is 0.048% and 2.383%, respectively, when the maximum inertia load is installed. The THD of the angular acceleration below a frequency of 1400 Hz is less than 0.02%, which is far below 2% of the exciter, as required by the ISO standard.

The above results of the calibration experiment indicate that the excitation system performs well in the passband of the MHD micro-angular vibration sensor under full-load inertia.

#### 3.2.2. Performance Experiments for the Homodyne Laser Interferometric Calibration System

The measurement range and repeatability of the homodyne laser interferometric calibration system need to be tested. Under non-reference conditions, the different angular velocity values that are required to calibrate the sensor can only be obtained by varying the amplitude of the angular vibration excitation. The measurement range and frequency characteristics of the calibration system are crucial for calibrating the angular velocity.

**Figure 14:**
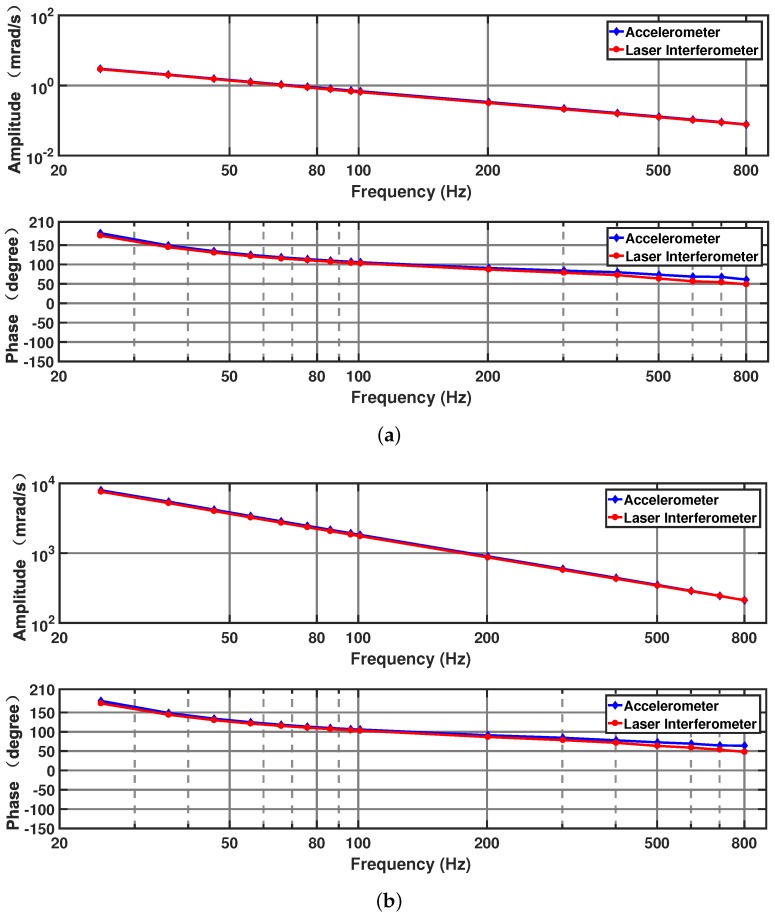
Frequency response of angular velocity under different angular excitation voltages (**a**) when the angular excitation voltage is 0.003 V and (**b**) when the angular excitation voltage is 8 V.

It was experimentally determined that the laser interferometer can precisely measure the angular velocity of the mechanism when the amplitude of the angular excitation voltage is between 0.003 and 8 V. Figure 14 presents the amplitude–frequency and phase–frequency curves for an excitation voltage amplitude of 0.003 and 8 V, respectively, with the corresponding output signal of the accelerometer plotted as a reference. The laser interferometer calibration system achieves a maximum calibratable angular velocity amplitude of 7590 mrad/s at 25 Hz and a minimum calibratable angular velocity amplitude of 0.076 mrad/s at 800 Hz. In this situation, the angle value corresponding to the maximum angular velocity does not exceed 3${}^{\circ }$, which satisfies the measurement conditions of the system. The amplitude–frequency curve trend of the laser interferometer is close to that of the accelerometer, which proves the reliability of the laser interferometric calibration process. The need to calibrate the angular vibration table is also proved since the amplitude and phase of the two still differ to some extent.

Experiments were carried out at different frequencies and different excitation voltage amplitudes. The angular velocity of the calibration system was repeatedly measured, and specific error indicators were analyzed. As presented in Table 5, experiments were performed at 36, 101, and 601 Hz, with the excitation voltage set to 0.04 V. The amplitude and phase repeatability error of the output angular velocity were calculated as $s=\sqrt{\frac{\sum_{i=1}^{n}{\left(\delta_{i}-\bar{\delta }\right)}^{2}}{n-1}}$, where $\delta_{i}$ is the *i*th measurement value, $\bar{\delta }$ is the average value, and *n* is the number of measurements.

The amplitude repeatability errors from five experiments at three frequencies (36, 101, and 601 Hz) are $1.924\times 10^{-3}$, $4.471\times 10^{-4}$, and $4.765\times 10^{-5}$ mrad/s, respectively, and the phase repeatability errors are $0.117^{\circ }$, $0.407^{\circ }$, and $1.096^{\circ }$. As the frequency increases and the angular velocity amplitude decreases, the repeatability error tends to increase.

The results above indicate that the homodyne laser interferometric calibration system excels in its ability to measure the micro-amplitude and wideband angular vibration, and it has great repeatability.

#### 3.2.3. Complex Sensitivity Calibration Experiments of the MHD Micro-Angular Vibration Sensor

In the calibration experiments that assessed the complex sensitivity parameter, the MHD micro-angular vibration sensor and angular retroreflector were fixed together to the top surface of the angular vibration table.

**Figure 15:**
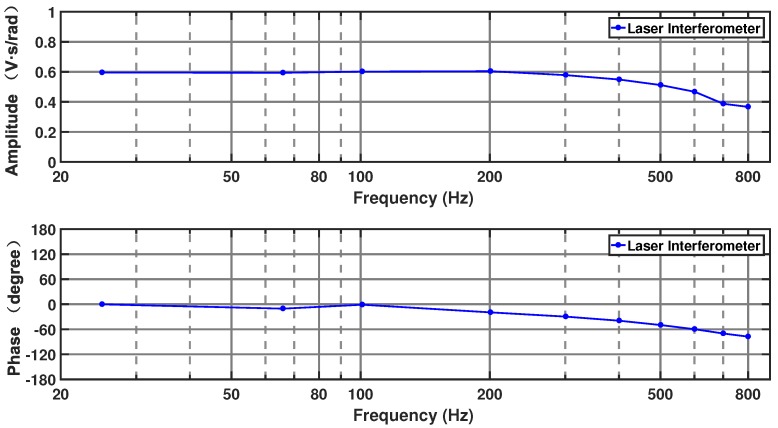
Frequency response of the complex sensitivity of the MHD micro-angular vibration sensor.

The curve in Figure 15 shows the sensitivity of the MHD micro-angular vibration sensor as it varies with frequency for an excitation voltage set to 1 V. For the frequencies from 25 to 200 Hz, the amplitude of the sensitivity is approximately 0.60 V/(rad/s), and the phase shift is from $0^{\circ }$ to $-15^{\circ }$. For the frequencies from 200 to 800 Hz, the amplitude of the sensitivity approximately ranges from 0.37 to 0.60 V/(rad/s), and the phase shift is from $-15^{\circ }$ to $-63^{\circ }$.

The repeatability of the sensitivity within the passband of the MHD micro-angular vibration sensor was verified by carrying out every ten repetitive experiments at 46, 201, and 401 Hz. Table 6 lists the complex sensitivity data of the repetitive experiments after processing. The maximum repeatability error of the sensitivity amplitude and the phase shift of the three frequencies are $7.20\times 10^{-4}$ V/(rad/s) and $0.720^{\circ }$. It can be concluded that the repeatability error increases to a small extent as the angular vibration frequency increases.

**Figure 16:**
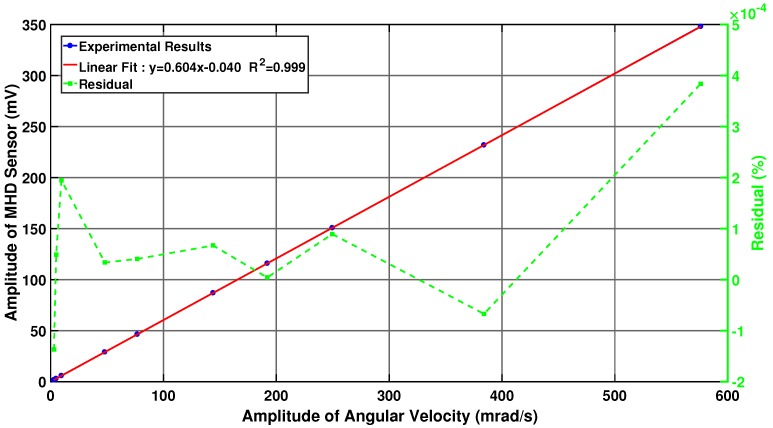
Sensitivity nonlinearity calibrating experiments of the MHD micro-angular vibration sensor.

The sensitivity nonlinearity of the MHD micro-angular vibration sensor was obtained for the variation in the angular velocity. As shown in Figure 16, the angular velocity amplitude and the sensor output voltage were measured by setting different excitation voltages at a frequency of 200 Hz. The data were fitted and calculated by $E=\pm \frac{\Delta_{\max}}{Y_{FS}}\times 100\%$, where $\Delta_{\max}$ is the maximum deviation of the sensor output voltage between the actual curve and the fitting line, and $Y_{FS}$ is the full-scale value of the sensor output voltage. The calculated nonlinearity error of the sensor is 0.022%.

#### 3.2.4. Uncertainty Evaluation of the System

This part presents the evaluation of the expanded uncertainties of the reference sensitivity amplitude and phase shift.

The mathematical models of the sensitivity amplitude *S* and phase shift ΔΦ are established in Equations (16) and (17).

(16) $$S=\frac{V_{MHD}}{\Omega }=\frac{V_{MHD}}{2\pi f\cdot \theta }$$

(17) $$\Delta \Phi =\varphi_{MHD}-\varphi_{\Omega }$$

where $V_{MHD}$ and $\varphi_{MHD}$ are the voltage amplitude and phase of the sensor output, respectively, θ and $\varphi_{\Omega }$ are the angle amplitude and angular velocity phase measured by the laser interferometer, and *f* is the frequency of the angular vibration.

According to the mathematical model, the amplitude uncertainty is evaluated by using the relative synthetic standard uncertainty $u_{c,rel}\left(s\right)$, as provided in Equation (18). Three components related to the uncertainty of the amplitude are $u_{V,rel}$, the output voltage measurement uncertainty of the MHD micro-angular vibration sensor; $u_{\theta ,rel}$, the output angle measurement uncertainty of the laser interferometry system; and $u_{f,rel}$, the measurement uncertainty of the angular vibration frequency.

(18) $$u_{c,rel}\left(s\right)=\sqrt{\sum_{i=1}^{N}{\left[c_{i}u\left(x_{i}\right)\right]}^{2}}=\sqrt{\sum_{i=1}^{N}{\left[\left|\frac{\partial s}{\partial x_{i}}\right|u\left(x_{i}\right)\right]}^{2}}=\sqrt{u_{V,rel}^{2}+u_{\theta ,rel}^{2}+u_{f,rel}^{2}}$$

where $u(x_{i})$ is the standard uncertainty corresponding to the *i*th input component $x_{i}$, and $c_{i}$ is the sensitivity coefficient of the *i*th standard uncertainty.

The sources of uncertainty for each the amplitude uncertainty component are listed in Table 7. The measurement uncertainty is classified into Type A and Type B according to the type of evaluation method. In Table 7, Type A uncertainty is obtained by repeating the experiments several times under the same conditions, and Type B uncertainty is obtained from experience, equipment calibration certificates, and papers, among other sources. The calculated relative standard uncertainty of the amplitude is $u_{c,rel}\left(s\right)=0.019\%$.

The sources of uncertainty for the phase shift of the reference sensitivity include the uncertainty introduced by the synchronization of data acquisition $u_{1}\left(\varphi \right)={\left(6\times 10^{-4}\right)}^{\circ }$, the uncertainty introduced by vibration interference $u_{2}\left(\varphi \right)=0.0577^{\circ }$, the uncertainty introduced by sensor output voltage noise $u_{3}\left(\varphi \right)={\left(1.4\times 10^{-5}\right)}^{\circ }$, the uncertainty introduced by photoelectric signal noise $u_{4}\left(\varphi \right)=0.0289^{\circ }$, and the uncertainty introduced by measurement repeatability error of the phase shift $u_{5}\left(\varphi \right)=0.618^{\circ }$. Therefore, the relative standard uncertainty of the phase shift can be calculated as $u_{c,rel}\left(\varphi \right)=0.621^{\circ }$.

If the inclusion factor *k* is 2, then the expanded uncertainties of the sensitivity amplitude and phase shift when the system calibrates the MHD micro-angular vibration sensor are 0.04% and $1.2^{\circ }$, respectively.

## 4. Conclusions

In the proposed system, the absolute calibration device is based on the combination of a homodyne laser interferometer and an angular retroreflector. Sinusoidal excitation with a single frequency is generated by the motor-type angular vibration table, and the output signals from the MHD micro-angular vibration sensor and laser interferometer are collected at the same time. In the end, both the interference signal and the MHD output voltage signal can be processed with the sinusoidal approximation method, and the amplitude and phase shift of sensor sensitivity can be obtained using the software. There is little error within a rotation range of $\pm 3^{\circ }$, and this method can effectively avoid errors due to the installation of the angular retroreflector. The experimental results indicate that the frequency measurement range of the laser interferometric calibration system can be as high as 800 Hz, and the angular velocity amplitude has a measurement range of 0.076–7590 mrad/s. In particular, the system can accurately calibrate the complex sensitivity of the MHD micro-angular vibration sensor with small angular vibration and a wide frequency range, and it shows high repeatability and linearity. The expanded uncertainties of the amplitude and phase shift are 0.04% and $1.2^{\circ }$ (k=2), respectively.

However, several problems in this system remain:

1. The evaluated amplitude uncertainty is much less than the international standard of 1%, while the phase shift uncertainty is larger than $1^{\circ }$. This is possibly caused by the complex vibration mode of the mechanical structure for small-amplitude and high-frequency vibration. Although the mechanical clamp was designed to reduce the barycenter and increase the structural stiffness to the greatest extent possible, the disequilibrium of the table load inertia and the mechanical connection can also lead to the desynchronization of the vibration state between the sensor and the retroreflector. This has become a limiting factor in micro-angular vibration calibration.
2. The homodyne interferometry method used in the system could be easily influenced by air turbulence and temperature differences because of the analysis of the direct current signal. In fact, this issue can be avoided when a heterodyne interferometer is used.
3. The proposed calibration method for the MHD micro-angular vibration sensor has not achieved the micro-radian scale.
4. The system currently uses the angular acceleration measured by the accelerometer to compare with the system output signal, which is unilateral. After solving the other problems in subsequent work, high-precision standard sensors will be needed to verify the accuracy of the system measurement results.
5. The system contains too many components, and its volume and weight should be reduced. Miniaturization needs to be considered for actual use.
6. The accuracy of the calibration system may be affected by the vibration of the spacecraft itself; this requires passive or active damping measures.

Future research will probably aim to solve the vibration mode problem. It is expected that the system will reach higher accuracy and that the dynamic calibration method will be applicable to other micro-angular vibration sensors.

## Figures and Tables

**Table 1:** Absolute calibration methods based on a laser interferometer.

Author	Characteristics of Methods	Key Parameters	Advantages and Disadvantages	Priority
PTB in 1998	Diffraction grating; Differential interference	Amplitude and phase expansion uncertainty: 0.3% and 0.2${}^{\circ }$	Difficult to guarantee the manufacturing accuracy of grating; Easy to introduce an installation error of 2 μm; Ability to measure large rotation angles.	★★
CIMM in 2006	Diffraction column grating; Heterodyne interferometer	Frequency range: 0.1~200 Hz; Amplitude and phase uncertainty: 1% and 1${}^{\circ }$	Coverage frequency of system is small.	★★
KRISS in 2006	Angle prism; Homodyne interferometer	Angle resolution: 2.55 × 10${}^{-8}$ rad; Angular velocity measuring ability: 100 rad/s; Relative standard uncertainty: 0.01~0.09%.	Suitable for measuring large rotation angles.	★
Xue JF. in 2014	Diffraction grating; Heterodyne interferometer; Differential measurement	Angle resolution: 0.02″.	Lateral vibration effects reduced by differential measurement.	★★★
Zhang X. in 2016	Ring laser; Differential measurement; Heterodyne interferometer	Frequency range: 1000 Hz; Sensitivity resolution: 0.0001″/Hz${}^{1/2}$	Size of the system is too large.	★
ISO Regulation: Amplitude and phase shift uncertainties of sensor sensitivity: 1% and 1${}^{\circ }$; THD of power amplifier and angular vibration exciter: ≤2%.				

**Table 2:** Relative calibration methods.

Methods	Principles	Advantages and Disadvantages	Priority
MEMS gyroscope	Angular momentum	Mature technology;	★
Fiber optic gyroscope	Sagnac effect	Low-precision and narrow-band commercial gyroscopes;	
Laser gyroscope	Sagnac effect	Difficult to obtain and expensive for high-precision gyroscope.	
Time grating sensor	”Time–space” effect	Angle resolution: 0.02”; Accuracy of calibration system: <2”; Poor high-frequency performance: ±8.2” accuracy at 400 Hz.	★

**Table 3:** Other calibration methods.

Methods	Principles	Advantages and Disadvantages	Priority
Two linear accelerometers	Circular motion	Easy to operate; Large installation error.	★
Free-oscillation method	Torsion pendulum principle	Inconsecutive frequency; Complex operation	★
Self-calibratable rotary encoder	Equal division averaged method	Self-calibration; Easy to operate; Only suitable for medium-performance MEMS gyroscopes.	★

**Table 4:** Signal THD of the angular excitation table carrying different loads.

Frequency (Hz)	THD (%)					
	No Additional Load		One Disk		Two Disk	
	Motor	Angular	Motor	Angular	Motor	Angular
	Current	Acceleration	Current	Acceleration	Current	Acceleration
21	0.011%	0.040%	0.009%	0.021%	0.010%	0.017%
41	0.007%	0.011%	0.007%	0.010%	0.008%	0.011%
61	0.008%	0.008%	0.007%	0.010%	0.008%	0.011%
81	0.007%	0.010%	0.007%	0.012%	0.007%	0.012%
101	0.007%	0.011%	0.007%	0.013%	0.007%	0.013%
211	0.007%	0.024%	0.006%	0.027%	0.007%	0.026%
411	0.008%	0.063%	0.007%	0.061%	0.008%	0.059%
611	0.014%	0.102%	0.014%	0.097%	0.015%	0.093%
811	0.024%	0.138%	0.024%	0.136%	0.024%	0.118%
1011	0.035%	0.172%	0.035%	0.164%	0.034%	0.135%
1211	0.041%	0.200%	0.041%	0.180%	0.042%	0.148%
1411	0.047%	0.236%	0.046%	0.213%	0.046%	0.268%
1611	0.048%	0.258%	0.048%	0.204%	0.048%	0.219%
1811	0.049%	0.287%	0.048%	0.607%	0.048%	0.513%
2011	0.047%	0.340%	0.047%	0.282%	0.045%	2.383%

**Table 5:** Results of repeatability experiments for angular velocity calibration.

Number	Frequency = 36 Hz		Frequency = 101 Hz		Frequency = 601 Hz	
	Amplitude	Phase	Amplitude	Phase	Amplitude	Phase
	(mrad/s)	(${}^{\circ }$)	(mrad/s)	(${}^{\circ }$)	(mrad/s)	(${}^{\circ }$)
1	26.397	144.453	8.835	102.891	1.458	57.466
2	26.400	144.396	8.835	102.313	1.352	59.502
3	26.401	144.605	8.835	102.373	1.458	59.426
4	26.402	144.684	8.834	102.940	1.461	59.640
5	26.401	144.493	8.835	103.274	1.457	57.590
Mean	26.400	144.526	8.835	102.758	1.437	58.725
Standard Deviation	1.924 $\times \;10^{-3}$	0.117	4.471 $\times \;10^{-4}$	0.407	4.765 $\times \;10^{-2}$	1.096

**Table 6:** Sensitivity repeatability data of the MHD micro-angular vibration sensor.

	Frequency = 46 Hz		Frequency = 201 Hz		Frequency = 401 Hz	
	Amplitude	Phase	Amplitude	Phase	Amplitude	Phase
	(V/(rad/s))	(${}^{\circ }$)	(V/(rad/s))	(${}^{\circ }$)	(V/(rad/s))	(${}^{\circ }$)
Mean	0.610	1.551	0.604	164.650	0.548	147.691
Standard Deviation	6.23 $\times \;10^{-5}$	0.119	3.00 $\times \;10^{-4}$	0.396	7.20 $\times \;10^{-4}$	0.720

**Table 7:** Relative standard uncertainty components of the sensitivity amplitude.

	Source of Uncertainty	Distribution	Type of Evaluation Method	Relative Standard Uncertainty Value
$u_{V,rel}$	A/D digits of the data acquisition card	Uniform	B	$u_{1}\left(V\right)=0.009\%$
	Voltage noise of sensor	Uniform	B	$u_{2}\left(V\right)=0.003\%$
	Repeatability error of voltage amplitude measurement	Normal	A	$u_{3}\left(V\right)=0.003\%$
	Vibration interference	Normal	B	$u_{4}\left(V\right)=0.0003\%$
$u_{\theta ,rel}$	Principle error of chord length replacing arc length	Uniform	B	$u_{5}\left(\theta \right)=0.006\%$
	Mounting error of optical device	Uniform	B	$u_{6}\left(\theta \right)=0.009\%$
	Noise of photoelectric signal	Uniform	B	$u_{7}\left(\theta \right)=0.0002\%$
	Vibration interference	Normal	B	$u_{8}\left(\theta \right)=0.0003\%$
	Manufacturing deviation of angular retroreflector	Uniform	B	$u_{9}\left(\theta \right)=0.011\%$
	Change in air refractive index	Uniform	B	$u_{10}\left(\theta \right)=0.003\%$
	Repeatability error of angle measurement	Normal	A	$u_{11}\left(\theta \right)=0.001\%$
$u_{f,rel}$	Accuracy of frequency	Normal	B	$u_{12}\left(f\right)=0.001\%$

## Abbreviations

The following abbreviations are used in this manuscript:

MHD	Magneto-Hydro-Dynamics
MIRU	Magnetohydrodymics Inertial Reference Unit
LLCD	Lunar Laser Communication Demonstration
NASA	National Aeronautics and Space Administration
OARE	Orbital Acceleration Research Experiment
IMU	Inertial Measurement Unit
MEMS	Microelectromechanical System
PTB	Physikalisch-Technische Bundesanstalt
ISO	International Organization for Standardization
CIMM	Changcheng Institute of Metrology and Measurement
KRISS	Korea Research Institute of Standards and Research
NIMJ	National Institute of Metrology of Japan
THD	Total Harmonic Distortion
